# Recurrent Kikuchi-Fujimoto Disease Masquerading as Lymphoma Successfully Treated by Anakinra

**DOI:** 10.7759/cureus.11655

**Published:** 2020-11-23

**Authors:** Beenish Faheem, Vinod Kumar, Hamdallah Ashkar, FNU Komal, Yasmeen Sultana

**Affiliations:** 1 Internal Medicine, St. Joseph's University Medical Center, Paterson, USA; 2 Oncology, St. Joseph's University Medical Center, Paterson, USA; 3 Hematology/Oncology, St. Joseph's University Medical Center, Paterson, USA

**Keywords:** kikuchi-fujimoto disease, kikuchi-fujimoto disease and systemic lupus erythematosus, kfd with diffuse lymphadenopathy, rare cause of diffuse lymphadenopathy, kikuchi-fujimoto disease exaggerated t-cell response

## Abstract

Kikuchi-Fujimoto disease (KFD), also known as histiocytic necrotizing lymphadenitis, is a rare and benign disease that usually presents in middle-aged women of Oriental-Asian ethnicity. This condition was described in Japan for the first time in 1972. Though the clinical course is benign, KFD has been misdiagnosed as malignancy (e.g. lymphoma) or infection. The most common presentation of KFD is with localized or generalized lymphadenopathy, fever, fatigue, weight loss, hepatosplenomegaly, and rash. A definitive diagnosis of KFD can be made by excisional lymph node biopsy, as immunohistochemical analysis is necessary. We present here an interesting case of a 20-year-old Hispanic female who was diagnosed with KFD who failed therapy with steroids and was subsequently treated successfully with the interleukin-1 (IL-1) inhibitor - anakinra.

## Introduction

Kikuchi-Fujimoto disease (KFD), also known as Kikuchi histiocytic necrotizing lymphadenitis, was originally described in young women and is a rare, benign condition of unknown cause. KFD is usually characterized by cervical lymphadenopathy, generalized lymphadenopathy, hepatomegaly, splenomegaly, fever, and weight loss. KFD can mimic lymphoma and other lymphoproliferative disorders and can only be differentiated via immunohistochemistry. The pathogenesis of KFD remains unclear but the virally-induced immune response is the most commonly proposed mechanism for etiology [1].

Clinical presentation, disease course, and histologic changes suggest an immune response of T cells and histiocytes to an infectious agent. Numerous inciting agents have been proposed, including Epstein-Barr virus (EBV), cytomegalovirus (CMV), human herpesvirus 6 (HHV6), human herpesvirus 8 (HHV8), human immunodeficiency virus (HIV), parvovirus B19, paramyxoviruses, parainfluenza virus, Yersinia enterocolitica, and Toxoplasma gondii [1,2].

KFD is usually treated with steroids and resolves within one to four months [1]. Often it may spontaneously remit without treatment and with no recurrence.

## Case presentation

We report a case of a 20-year-old Hispanic female with a past medical history of rheumatoid arthritis (RA) and juvenile idiopathic arthritis (JIA) diagnosed at 18 years.

The patient presented to the emergency department with complaints of subjective fevers of four to six weeks duration, intermittent, and mostly occurring at night. Her fever was associated with a rash on bilateral hands with left wrist pain and swelling. The patient also complained of swollen lymph nodes bilaterally in her neck, axilla, and inguinal regions, along with unintentional weight loss of 20 pounds over two months associated with night sweats.

On physical examination, the patient was found to have a temperature of 102 degrees Celsius, heart rate (HR) 112 beats/min, blood pressure (BP) 110/70 mmHg, respiratory rate: 18/min. Her complete blood count revealed white blood cell count (WBC) of 2.3 x10^9^/L (normal range: 4.5-11.0 x 10^9^/L) with lymphocytes count of 1.2 x10^3^/mm^3^, (normal range: 1,000-4,000/mm^3^) erythrocyte sedimentation rate (ESR): 110 mm/hr (normal range: 0-22 mm/hr), C-reactive protein (CRP): 80 mg/L (normal range: <10 mg/L). The patient’s HIV, hepatitis panel, EBV, and CMV results were all negative.

**Figure 1:**
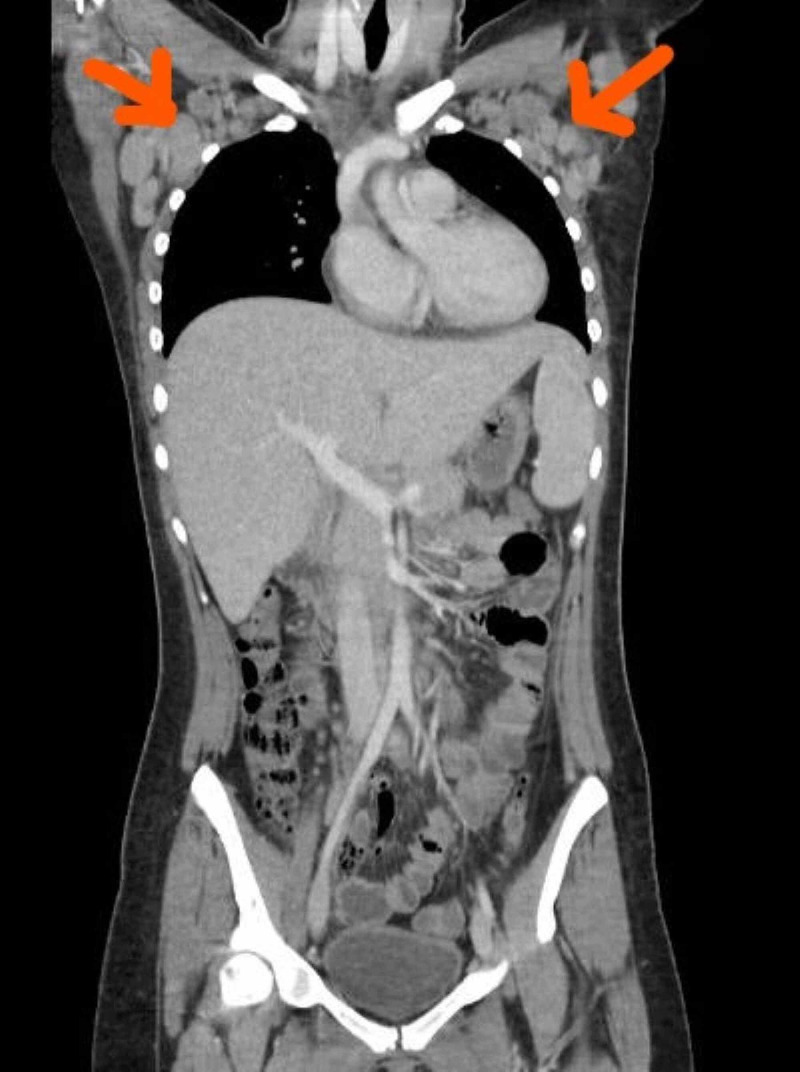
There is extensive axillary lymphadenopathy with a conglomeration of all of the lymph nodes in the right axilla measuring approximately 6.5 cm. The largest lymph node within this conglomeration is 3.6 cm on the right and 3.3 cm on the left.

**Figure 2:**
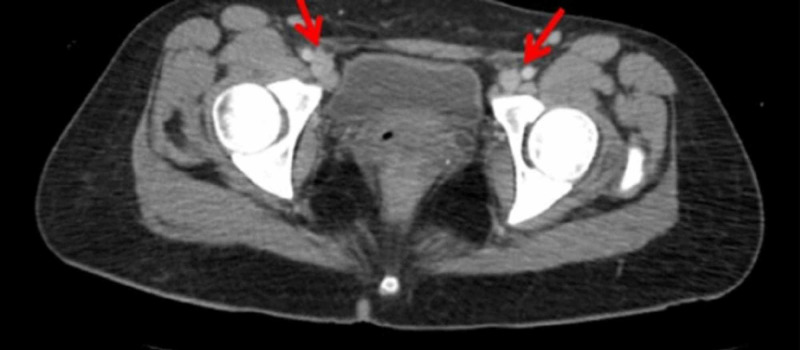
Kikuchi-Fujimoto disease with extensive bilateral inguinal lymphadenopathy (red arrows).

The computer tomography (CT) imaging of her chest, abdomen, and pelvis revealed cervical, mediastinal, and inguinal lymphadenopathy along with hepatomegaly, and splenomegaly (Figures 1-2).

Based on history, physical examination, routine labs, and imaging studies, the initial working diagnosis was Hodgkin's disease. We decided to do an excisional cervical lymph node biopsy.

**Figure 3:**
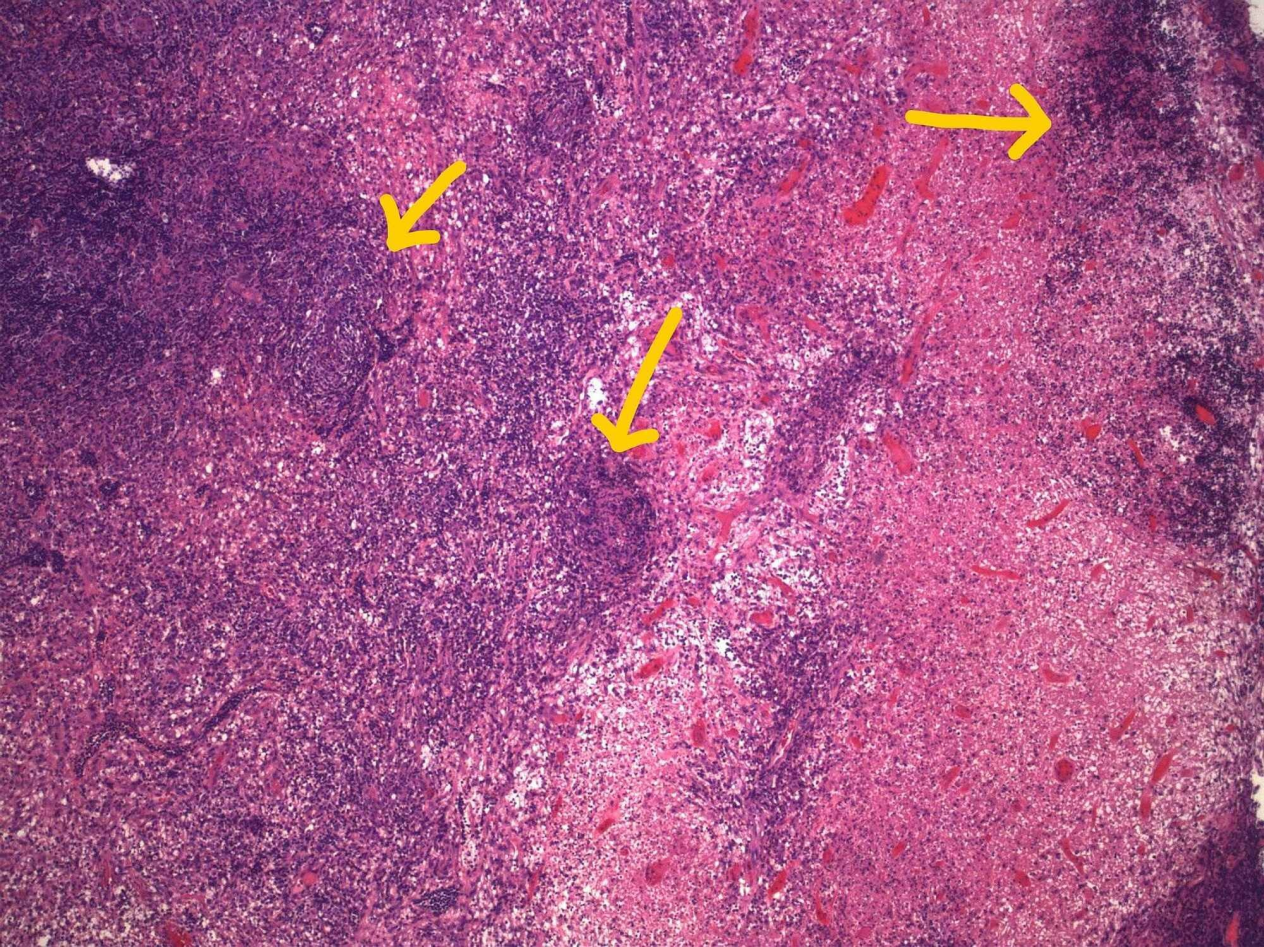
Excisional cervical lymph node biopsy section at low power (hematoxylin and eosin x40) demonstrating patchy areas of necrosis, histiocytosis with abundant cytoplasm, and paracortical hyperplasia

**Figure 4:**
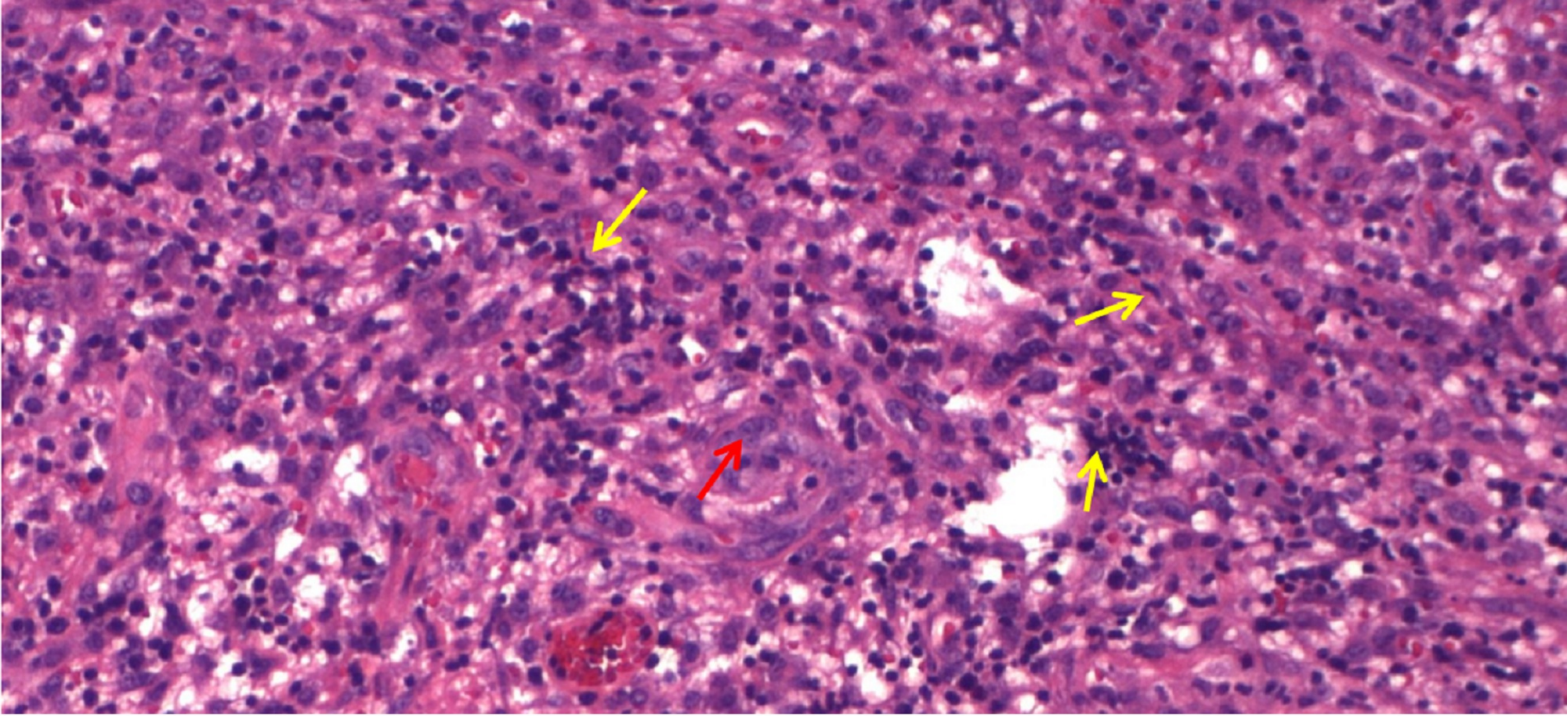
A high-power view (hematoxylin and eosin x400) displaying focal necrosis surrounded by karyorrhectic debris (red arrow), plasmacytoid lymphocytes, and characteristic crescentic histiocytes (yellow arrows) among scattered apoptotic cells.

The excisional biopsy was consistent with KFD disease, demonstrating a necrotizing pattern, and the patient was subsequently started on a long course of steroids (Figures 3-4). The patient initially responded well for a few months but had multiple relapses on steroids. Her repeat labs revealed persistent leukocytosis with high inflammatory markers like ESR and CRP. Her repeat CT scan showed an increase in the size of her cervical, mediastinal, and inguinal lymphadenopathy and an increase in the size of her spleen.

Based on these findings, the steroids were tapered and discontinued. The patient was subsequently started on the second line treatment drug, anakinra (interleukin-1 (IL-1)). She demonstrated good clinical response and has been in remission for three years now with the newer agent, anakinra.

## Discussion

KFD was first described by Kikuchi and Fujimoto in Japan in 1972, as histiocytic necrotizing lymphadenitis, a self-limiting syndrome, with distinct histological features [2]. The clinical symptoms include, but are not limited to, cervical lymphadenopathy, hepatosplenomegaly, fever, malaise, arthralgias, and weight loss. KFD is most commonly seen among middle-aged adults. A female preponderance of KFD is reported in the literature. Although KFD has a worldwide distribution, there seems to be a higher prevalence among Japanese and people of other oriental-Asiatic regions [2]. 

The causes of KFD continues to be a challenge with much speculation, an autoimmune or a viral cause with an exaggerated T-cell-mediated immune response is usually suggested [2]. There have been reports suggesting Yersinia enterocolitica and Toxoplasma gondii as possible bacteria causing KFD but subsequent studies failed to support these hypotheses as the features of lymphadenitis associated with these microorganisms differ clearly from those of KFD [2]. It is hypothesized that the etiology may be post-viral or linked to an autoimmune disease, such as systemic lupus erythematosus (SLE). A host of viruses along with EBV and CMV have been considered in the pathogenesis of KFD; however, serologic testing for their antibodies has revealed negative results consistently and no viral particles have been isolated [2]. Similarly, SLE and other autoimmune disorders have been hypothesized to be culprits for KFD; however, serologic testing for antinuclear antibodies and the rheumatoid factor has consistently resulted in the negative [2]. There is some suggestion that given the histologic, ultrastructural, and immunohistochemistry findings that a hyper-immune reaction toward various organisms may be causing KFD [2]. There may be an excessive T-cell-mediated immune response in genetically susceptible people to various nonspecific stimuli. A high incidence of particular human leukocyte antigen (HLA) class II genes has been reported in patients with KFD, particularly DPA1*01 and DPB1*0202 alleles, compared to healthy control subjects. Studies suggest that so far these genes have been either rare or absent among Caucasians but common among Oriental-Asian people, which may help explain the epidemiologic pattern [2].

There are no specific diagnostic laboratory tests to definitively diagnose KFD [3]. Elevated lactate dehydrogenase, neutropenia, lymphocytosis, leukopenia, abnormal liver enzymes, and elevated sedimentation rates have been reported. The diagnosis is based primarily on the lymph node biopsy in the setting of typical clinical symptoms. The histologic features include circumscribed and patchy necrosis bordered by macrophage, histiocytes, and lymphoid cells [3]. Furthermore, the histopathological diagnosis is characterized by three different patterns: proliferative, necrotizing, and xanthomatous [4]. Our patient’s biopsy revealed a necrotizing pattern. It is within the necrotizing lesions that plasmacytoid monocytes or plasmacytoid T-cells were present, stained with cluster of differentiation 68 (CD68), and it is this feature that is the hallmark histopathologic finding unique to KFD [2].

There is no specific treatment for patients with KFD; however, symptomatic management with anti-inflammatories and antipyretic therapies provides relief [3]. In severe cases, corticosteroids have been known to provide symptomatic relief. In some studies, hydroxychloroquine, alone or in combination with glucocorticoids, have shown benefit among symptomatic KFD patients. It is hypothesized that the anti-inflammatory effects coupled with the immunomodulatory effects of this treatment regimen, particularly on antigen presentation with protein degradation, is what explains its success and may potentially become a drug of choice for symptomatic KFD [5]. Typically symptoms resolve within six months [1]. KFD has been reported to relapse with a low recurrence rate ranging between 3%-4% [6]. In our patient, corticosteroids showed great benefit but only initially as she had multiple recurrences; therefore, anakinra was considered. Anakinra is a recombinant human IL-1 receptor inhibitor that blocks the downstream inflammatory actions of IL-1α and IL-1β [7]. There has been substantial evidence that IL-1 inhibitors have a strong steroid-sparing effect. It has shown therapeutic benefit in autoimmune diseases such as adult-onset Still disease (AOSD) and juvenile Still disease and is currently Food and Drug Administration (FDA) approved to treat RA and neonatal-onset multisystem inflammatory disease (NOMID). Given our patient's comorbidities of RA and JIA, we decided to use anakinra after she failed therapy with steroids. Once the IL-1 inhibitor was started, the patient demonstrated an excellent response and continues to be in remission now for almost three years. 

## Conclusions

KFD is a rare condition characterized as benign histiocytic necrotizing lymphadenitis. It is common among Asian people and its etiology is still not fully understood. It is hypothesized that the etiology may be post-viral or linked to autoimmune disease and tends to affect people younger than 40 years of age with a female preponderance. KFD most commonly presents with pyrexia, localized and generalized lymphadenopathy, night sweats, weight loss, hepatosplenomegaly, fatigue, and rash. KFD is definitively diagnosed via lymph node tissue biopsy. Although there is no specific treatment for KFD, its symptoms can be alleviated by using nonsteroidal anti-inflammatory drugs or, if severe, with corticosteroids. Some studies have shown benefit with biologics such as anakinra, an IL-1 inhibitor, and antimalarials such as hydroxychloroquine combined with steroids for relapsing KFD. It is important to recognize KFD as a potential cause of persistent and recurrent lymphadenopathy, as it can easily be mistaken for other causes of lymphadenopathy, including Hodgkin's lymphoma, autoimmune diseases such as SLE, infectious and inflammatory conditions. Appropriate recognition of KFD is important as it will help prompt the appropriate treatment course, with long-term follow-up as relapses can occur, such as in our patient reported in this case. Here anakinra showed benefit in recurrent KFD and allowed for remission of disease course.
